# Examining the impact of chronic diseases on activities of daily living of middle-aged and older adults aged 45 years and above in China: a nationally representative cohort study

**DOI:** 10.3389/fpubh.2023.1303137

**Published:** 2024-02-14

**Authors:** Zhonghua Ai, Churou Tang, Xuan Wen, Karthikesu Kartheepan, Songyuan Tang

**Affiliations:** ^1^Institute of Health Studies, School of Public Health, Kunming Medical University, Kunming, Yunnan, China; ^2^Department of Biology, University of Rochester, Rochester, NY, United States; ^3^School of Public Health, Kunming Medical University, Kunming, Yunnan, China; ^4^Department of Primary Health Care, Faculty of Health-Care Sciences, Eastern University, Chenkalady, Sri Lanka

**Keywords:** chronic diseases, activities of daily living, middle-aged and older adults, cohort study, China

## Abstract

**Background:**

China has by far one of the fastest-aging populations in the world. Increasing age is often accompanied by an increasing prevalence of chronic diseases and impaired Activities of Daily Living (ADL). The aim of this study was to analyze the effects of chronic diseases on ADL in Chinese middle-aged and older adults and to provide a scientific basis for delaying the impairment of ADL and prolonging the self-care life expectancy of middle-aged and older adults.

**Methods:**

This investigation utilized the survey information of 10,096 middle-aged and older adults from the China Health and Aging Tracking Survey (CHARLS) of 2011 as baseline data, then followed up this cohort until 2018, and performed multifactorial analyses using Cox proportional risk models to explore the strength of the associations between chronic diseases and the risk of impaired ADL in middle-aged and older adults.

**Results:**

Among the middle-aged and older adult population, the presence of hypertension was associated with a 38% higher risk of impaired ADL compared to those without the condition (*HR* = 1.38,95% *CI*:1.24–1.54); the involvement of heart disease was associated with a 27% higher risk of impaired ADL compared to those without the condition (*HR* = 1.27,95% *CI*:1.10- 1.46); the existence of arthritis was associated with a 38% higher risk of impaired ADL in middle-aged and older adults compared to those without arthritis (*HR* = 1.38,95% *CI*:1.25–2.08); additionally, the risk of impaired ADL with one or ≥ 2 chronic diseases was increased by 34% (*HR* = 1.34, 95% *CI*:1.18–1.52) and 84% (*HR* = 1.84, 95% *CI*:1.63–2.08) in middle-aged and older adult individuals, respectively.

**Conclusion:**

Hypertension is a risk factor for impaired ADL at any age in the subjects of this study. Examining the association between the number of chronic diseases and impairment in activities of daily living, it was revealed that the risk of ADL impairment increased with the number of chronic diseases in both the middle-aged (45–59 years) and older adult (60–74 years) groups.

## Introduction

Population aging has become a major global problem, and China has the world's largest older adult population and one of the fastest-aging populations in the world (1). In 2022, 14.9% of the country's population was 65 years of age or older. It is expected that by 2050, China's older adult population aged 65 and above will reach 382 million, accounting for 32.5% of the total population (2). As we age, all organs and functions of the human body weaken, and the ability to perform activities of daily living (ADL) declines.

ADL refers to the fundamental duties that are necessary for a person to carry out on a daily basis. These activities include using the restroom, eating, clothing, grooming, walking, and taking a bath. ADL impairment indicates a person's inability to perform these necessary tasks on their own, which exerts a heavy load on the person, their family, and society at large (3). As of 2021, approximately 16% of the world's population are affected by ADL impairment, with 34.4% of those aged 60 years and older affected (4). The ADL indicator has been officially recognized by the World Health Organization (WHO) and is widely used (5). There are many factors that influence ADL impairment in middle-aged and older adults, and previous studies have shown that low socioeconomic status, low education, cerebral hemorrhage (6), absence of a spouse, physical inactivity, low social activity, experience of falls, cognitive decline (7), being female, admission to a nursing home for ≥5 years, and impaired consciousness (8) are all factors contributing to ADL impairment.

However, there is a paucity of research on the relationship between chronic conditions and impaired ADL. A study published in 2023 noted that the negative impact of chronic diseases on the quality of life of older adults is not limited to physical health, but also involves psychological and social aspects (9). Approximately 70% of the total disease burden experienced by the Chinese population in 2019 was attributable to chronic diseases. The health damage caused by chronic diseases is immeasurable and has become the most important cause of death among Chinese residents, accounting for approximately 88.5% of deaths (10). Chronic diseases have demonstrated an aging trend in recent years. The high prevalence of chronic diseases aggravates the physical deterioration and daily life dysfunction of middle-aged and older adults, which in turn leads to a continuous decline in the self-care ability and quality of life of middle-aged and older adults, and imposes a heavy disease burden on individuals, families and society (11).

Few studies have been conducted both nationally and internationally to investigate the relationship between chronic diseases and impaired ADL, and the studies that have been carried out have utilized primarily cross-sectional data (12, 13). Limited studies have examined the impact of co-morbidities and chronic diseases interacting with other risk variables on ADL in middle-aged and older adults using cohort data. Therefore, this study utilized data from the China Health and Retirement Longitudinal Study (CHARLS) to analyze the effects of chronic diseases on ADL in middle-aged and older adults in China, using a retrospective cohort study design to provide a scientific basis for delaying the impairment of ADL and improving the quality of life in middle-aged and older adults.

## Materials and methods

### Data sources and sample

Data were obtained from the CHARLS database. The database is designed to study the issue of aging in China and to facilitate corresponding research. The CHARLS study used a multistage stratified random sample of 450 village-level units in 150 counties (districts) in 28 provinces (autonomous regions and municipalities directly under the central government). Survey weights were considered in all our analyses as follows. The product of the basic and adjusted weights was used to obtain a combined weight for each individual to represent the probability of its true occurrence in the aggregate. To make the data a better representation, the weighting considers a number of factors, including the sampling design, missingness, non-response rate, non-coverage, geographical location, gender, age, and socioeconomic characteristics (14). The ethical approval for the CHARLS project was received from the Ethics Review Committee of Peking University, and informed consent was obtained from all participants during the data collection.

**Figure 1:**
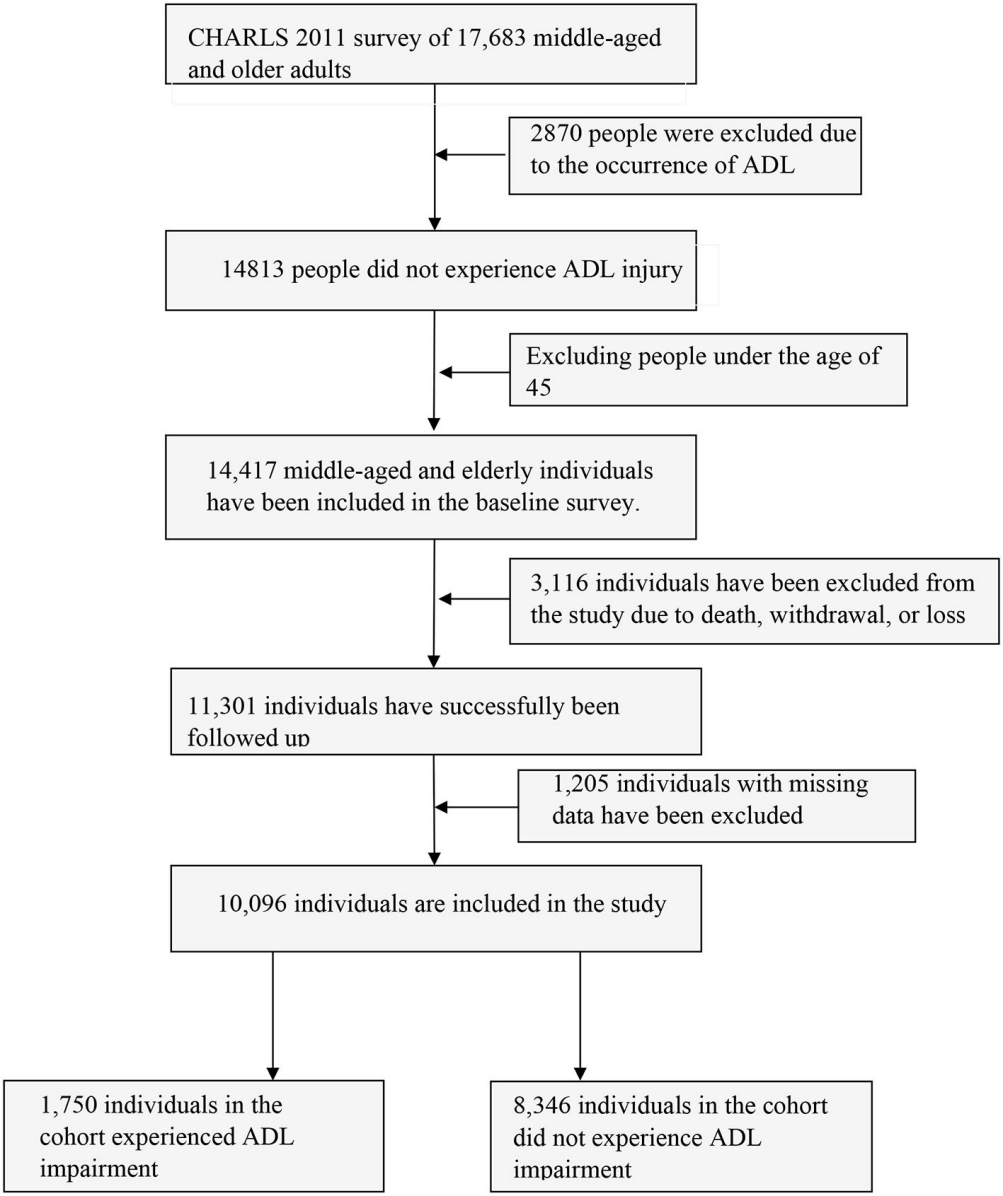
Sample screening process.

A total of 17,683 samples were collected for the survey at the 2011 baseline. After excluding 396 individuals under 45 years old and 2,870 who had already experienced ADL impairment at the time of the baseline survey, 14,417 middle-aged and older adults who were eligible for the study were ultimately included in the study. As a result of death, withdrawal, loss of visits, etc., the sample reported 11,301 individuals at the end of the follow-up in 2018. However, due to missing data and other factors, a valid sample of 10,096 individuals was finally included. Of these, 8,346 did not have an ADL loss, and 1,750 individuals in the group had an ADL injury. Prior to and during sample removal, there was no statistically significant difference between middle-aged and older adults with and without ADL injury within the cohort, indicating a low likelihood of bias from lost visits, data cleaning, and other reasons. Figure 1 depicts the particular sample screening procedure.

### Baseline survey

This study used a cohort study method, with 14,417 middle-aged and older adults aged ≥45 years obtained from the 2011 survey as the baseline population. The exclusion criteria for the study population were: respondents with impaired ADL at the time of the baseline survey; and age under 45 years. Inclusion criteria were: respondents did not meet the exclusion criteria; no key variables were missing. The study variables of this survey are basic demographic factors (age, gender, region, residence, marital status, education level), lifestyle that may be associated with impaired ADL (smoking status, alcohol consumption status, social activity status) (15), personal health status (ability to perform activities of daily living, physical pain status, fall status), and chronic disease prevalence (cardiovascular disease, respiratory disease, digestive disease, metabolic disease, neurological disease, arthritis or rheumatism, and kidney disease). Data on these study variables were collected using a validated, reliable questionnaire on subjects previously diagnosed with chronic diseases by health-care providers (16).

### Treatment of study factors

According to WHO standards, middle-aged and older adult individuals were divided into three groups for this study: middle-aged individuals 45–59 years old, young older adult individuals 60–74 years old, and older adult individuals 75 years of age and older. Also investigated was how various groups' ADL was affected by their status with chronic diseases. Hypertension, dyslipidemia, heart disease, gastric or digestive diseases, arthritis, and other conditions with prevalence in the top five were chosen as the study factors based on the chronic disease parity of middle-aged and older adults, as reported in the 2018 CHARLS data analysis report. The quantity of prevalence outside of these five categories of chronic diseases was used as the control number. In this study, the number of chronic diseases suffered by middle-aged and older adults was divided into three categories (0, 1, and 2). The majority of these individuals had several co-morbidities. Furthermore, there are significant differences in middle-aged and older adult health status between age groups.

### Follow-up survey

The ADL impairment status of the middle-aged and older adult cohorts was followed up in 2013, 2015, and 2018, respectively. The outcome events were categorized as “occurrence of ADL impairment” and “deletion.” Middle-aged and older adults who were lost during the follow-up, who died before the end of the follow-up without ADL impairment, and who did not have ADL impairment at the end of the last follow-up in 2018 were labeled as censored. The definition of impaired ability to perform activities of daily living includes using the restroom, eating, clothing, grooming, walking, and taking a bath. Each question is rated on a four-point scale (1: no difficulty). 2: difficulty but still can be completed. 3: difficulty and need help. 4: cannot be completed.). A total score equal to 6 is considered completely normal; a score greater than 6 represents a middle-aged or older adult person with varying degrees of ADL impairment. A score of 1 for a single question is considered normal, and a score of 2–4 is considered a decrease in functioning; if two or more scores are greater than or equal to 3, or if the total score is greater than or equal to 12, it represents a significant impairment of the ADL. Some studies have shown that the ADL scale has high reliability and validity in reflecting the mental and health status of Chinese older adults (15).

### Statistical analysis

In this study, the distribution of categorical variables was described using n (%), and the log-rank one-way test was used to analyze whether there was a statistical association between exposure factors and the risk of developing impaired ADL in the middle-aged and older adult population; multifactorial analysis using Cox proportional risk models to explore the strength of the association between hypertension, dyslipidaemia, heart disease, gastric or digestive disorders, arthritis, and the number of chronic illnesses suffered and the risk of impaired ADLs in middle-aged and older adults; Finally, the VIF variance inflation factor was used to test for variable covariance. All analyses were performed using the statistical software Stata 16.0, and a two-tailed *P* value < 0.05 was considered statistically significant.

## Results

Sixty-eight percent of the 10,096 sample population that was included in the survey was between the ages of 45 and 59, thirty-nine percent was between the ages of 60 and 74, and four percent was above the age of 75. The following factors were found by the log-rank test to be statistically significant in the difference between the ADL impaired group and the ADL intact group in the 45–59-year age group: gender, place of residence, marital status, education level, smoking, drinking, physical pain status, fall status, arthritis, heart disease, dyslipidemia, number and region of chronic diseases, and hypertension. Moreover, in the subgroup 60–74 years, hypertension, heart disease, stomach or digestive disease, arthritis, number and gender of chronic diseases, place of residence, marital status, education, smoking status, alcohol consumption status, social activity status, physical pain status, and fall status showed statistically significant differences between the ADL impaired group and the ADL intact group by the log-rank test. Besides, the ADL impaired and ADL intact groups differed statistically significantly in alcohol consumption status, gender, and hypertension in the 75 and over subgroup (Table 1).

**Table 1:** Descriptive analysis of demographic characteristics and baseline exposure factors in the middle-aged and older adult population (%).

**Variable**	**45–59 years (*****n =*** **6,241)**				**60**~**74 years (*****n =*** **3,420)**			
	**ADL impaired (*n =* 772)**	**ADL intact (*n =* 5,469)**	**χ^2^**	***P***	**ADL impaired (*n =* 813)**	**ADL intact (*n =* 2,607)**	**χ^2^**	***P***
**Region**								
Eastern China	222 (10.0)	2,000 (90.0)	19.75	< 0.001	246 (21.4)	903 (78.6)	5.82	0.054
Central China	269 (13.0)	1,797 (87.0)			280 (24.4)	870 (75.6)		
Western China	281 (14.4)	1,672 (85.6)			287 (25.6)	834 (74.4)		
**Sex**								
Male	261 (9.1)	2,616 (90.9)	53.55	< 0.001	337 (19.4)	1,402 (80.6)	37.68	< 0.001
Female	511 (15.2)	2,853 (84.8)			476 (28.3)	1,205 (71.7)		
**Place of residence**								
Rural	539 (4.0)	3,323 (86.0)	23.53	< 0.001	554 (26.2)	1,562 (73.8)	17.78	< 0.001
Urban	233 (9.8)	2,146 (90.2)			259 (19.9)	1,045 (80.1)		
**Marital status**								
Married	673 (12.1)	4,910 (87.9)	4.86	0.028	645 (22.9)	2,172 (77.1)	6.75	0.009
Divorced/widowed	99 (15.1)	559 (84.9)			168 (27.9)	435 (72.1)		
**Educational attainment**								
Illiterate	248 (20.4)	969 (79.6)	102.98	< 0.001	306 (30.1)	712 (69.9)	41.80	< 0.001
Did not finish primary school	134 (13.9)	827 (86.1)			178 (25.4)	522 (74.6)		
Primary and above	390 (9.6)	3,673 (90.4)			329 (19.3)	1,373 (80.7)		
**Smoking status**								
No	527 (13.5)	3,375 (86.5)	12.40	< 0.001	501 (25.4)	1,469 (74.6)	7.06	0.008
Yes	245 (10.5)	2,094 (89.5)			312 (21.5)	1,138 (78.5)		
**Alcohol consumption**								
No	559 (14.0)	3,435 (86.0)	27.60	< 0.001	611 (26.3)	1,710 (73.7)	25.98	< 0.001
Yes	213 (9.5)	2,034 (90.5)			202 (18.4)	897 (81.6)		
**Social activities**								
No	416 (14.1)	2,543 (85.9)	14.81	< 0.001	466 (27.1)	1,252 (72.9)	21.41	< 0.001
Yes	356 (10.9)	2,926 (89.1)			347 (20.4)	1,355 (79.6)		
**Physical pain**								
No	446 (9.8)	4,093 (90.2)	99.36	< 0.001	471 (19.2)	1,985 (80.8)	101.50	< 0.001
Yes	326 (19.2)	1,376 (80.8)			342 (35.5)	622 (64.5)		
**Falls**								
No	632 (11.5)	4,866 (88.5)	32.60	< 0.001	661 (22.8)	2,238 (77.2)	9.90	0.002
Yes	140 (18.8)	603 (81.2)			152 (29.2)	369 (70.8)		
**High blood pressure**								
No	598 (11.6)	4,551 (88.4)	15.51	< 0.001	524 (21.5)	1,910 (78.5)	23.45	< 0.001
Yes	174 (15.9)	918 (84.1)			289 (29.3)	697 (70.7)		
**Dyslipidaemia**								
No	692 (12.1)	5,043 (87.9)	6.01	0.014	720 (23.4)	2,352 (76.6)	1.86	0.172
Yes	80 (15.8)	426 (84.2)			93 (26.7)	255 (73.3)		
**Heart disease**								
No	671 (11.7)	5,084 (88.3)	34.41	< 0.001	674 (22.9)	2,273 (77.1)	9.55	0.002
Yes	101 (20.8)	385 (79.2)			139 (29.4)	334 (70.6)		
**Stomach or gastrointestinal disease**								
No	572 (11.6)	4,348 (88.4)	11.86	0.001	598 (22.1)	2,103 (77.9)	18.88	< 0.001
Yes	200 (15.1)	1,121 (84.9)			215 (29.9)	504 (70.1)		
**Arthritis**								
No	443 (9.9)	4,036 (90.1)	89.96	< 0.001	447 (19.6)	1,835 (80.4)	66.25	< 0.001
Yes	329 (18.7)	1,433 (81.3)			366 (32.2)	772 (67.8)		
**Number of chronic diseases**								
0	206 (8.2)	2,308 (91.8)	95.51	< 0.001	161 (16.0)	848 (84.0)	87.35	< 0.001
1	240 (12.4)	1,689 (87.6)			226 (21.0)	850 (79)		
> = 2	326 (18.1)	1,472 (81.9)			426 (31.9)	909 (68.1)		
**Variable**	**75 years and over (*****n** =* **435)**				**Add up the total (*****n** =* **10,096)**			
	**ADL impaired (*****n** =* **165)**	**ADL intact (*****n** =* **270)**	χ^2^	***P***	**ADL impaired (*****n** =* **1,750)**	**ADL intact (*****n** =* **8,346)**	χ^2^	***P***
**Region**								
Eastern China	65 (35.7)	117 (64.3)	0.87	0.647	533 (15.0)	3,020 (85.0)	23.40	< 0.001
Central China	48 (38.1)	78 (61.9)			597 (17.9)	2,745 (82.1)		
Western China	52 (40.9)	75 (59.1)			620 (19.4)	2,581 (80.6)		
**Sex**								
Male	66 (30.7)	149 (69.3)	9.45	0.002	664 (13.7)	4,167 (86.3)	83.28	< 0.001
Female	99 (45.0)	121 (55.0)			1,086 (20.6)	4,179 (79.4)		
**Place of residence**								
Rural	95 (37.9)	156 (62.1)	0.01	0.967	1,188 (19.1)	5,041 (80.9)	34.30	< 0.001
Urban	70 (38.0)	114 (62.0)			562 (14.5)	3,305 (85.5)		
**Marital status**								
Married	88 (34.7)	166 (65.3)	2.80	0.094	1,406 (16.3)	7,248 (83.7)	49.94	< 0.001
Divorced/widowed	77 (42.5)	104 (57.5)			344 (23.9)	1,098 (76.1)		
**Educational attainment**								
Illiterate	95 (40.4)	140 (59.6)	1.90	0.386	649 (26.3)	1,821 (73.7)	217.99	< 0.001
Did not finish primary school	19 (31.2)	42 (68.8)			331 (19.2)	1,391 (80.8)		
Primary and above	51 (36.7)	88 (63.3)			770 (13.0)	5,134 (87.0)		
**Smoking status**								
No	112 (40.4)	165 (59.6)	2.03	0.154	1,140 (18.5)	5,009 (81.5)	15.96	< 0.001
Yes	53 (33.5)	105 (66.5)			610 (15.5)	3,337 (84.5)		
**Alcohol consumption**								
No	133 (41.4)	188 (58.6)	6.38	0.012	1,303 (19.6)	5,333 (80.4)	71.59	< 0.001
Yes	32 (29.1)	82 (71.9)			447 (12.9)	3,013 (87.1)		
**Social activities**								
No	81 (39.9)	122 (60.1)	0.63	0.428	963 (19.7)	3,917 (80.3)	37.97	< 0.001
Yes	84 (36.2)	148 (63.8)			787 (15.1)	4,429 (84.9)		
**Physical pain**								
No	122 (35.8)	219 (64.2)	3.11	0.078	1,039 (14.2)	6,297 (85.8)	188.26	< 0.001
Yes	43 (45.7)	51 (54.3)			711 (25.8)	2,049 (74.2)		
**Falls**								
No	130 (36.8)	223 (63.2)	0.97	0.325	1,423 (16.3)	7,327 (83.7)	52.51	< 0.001
Yes	35 (42.7)	47 (57.3)			327 (24.3)	1,019 (75.7)		
**High blood pressure**								
No	101 (33.5)	200 (66.5)	7.95	0.005	1,223 (15.5)	6,661 (84.5)	83.29	< 0.001
Yes	64 (47.8)	70 (52.2)			527 (23.8)	1,685 (76.2)		
**Dyslipidaemia**								
No	157 (38.9)	247 (61.1)	2.08	0.149	1,569 (17.0)	7,642 (83.0)	6.58	0.01
Yes	8 (25.8)	23 (74.2)			181 (20.5)	704 (79.5)		
**Heart disease**								
No	141 (37.5)	235 (62.5)	0.22	0.64	1,486 (16.4)	7,592 (83.6)	58.43	< 0.001
Yes	24 (40.7)	35 (59.3)			264 (25.9)	754 (74.1)		
**Stomach or gastrointestinal disease**								
No	137 (37.7)	226 (62.3)	0.03	0.855	1,307 (16.4)	6,677 (83.6)	24.71	< 0.001
Yes	28 (38.9)	44 (61.1)			443 (21.0)	1,669 (79.0)		
**Arthritis**								
No	118 (36.9)	202 (63.1)	0.57	0.449	1,008 (14.2)	6,073 (65.8)	158.85	< 0.001
Yes	47 (40.9)	68 (59.1)			742 (24.6)	2,273 (75.4)		
**Number of chronic diseases**								
0	54 (34.6)	102 (65.4)	1.47	0.479	421 (11.4)	3,258 (88.6)	219.52	< 0.001
1	47 (37.9)	77 (62.1)			513 (16.4)	2,616 (83.6)		
≥ 2	64 (41.3)	91 (58.7)			816 (24.8)	2,472 (75.2)		

Furthermore, a Variance Inflation Factor (VIF) test was conducted to assess the multicollinearity between the independent variables in the model. The VIF value measures the degree of multicollinearity between the independent variables, and in general, a VIF value of < 10 means that it is acceptable (17). In Table 2, the mean VIF values for the four models are 2.35, 2.13, 2.12, and 2.23 from left to right, and the VIF values for all variables are < 5, which indicates that there is no multicollinearity among the variables. In Table 3, the mean values of VIF for the four models are 2.63, 2.38, 2.33, and 2.48 from left to right, and the VIF values of all variables are < 6, which indicates that there is no multicollinearity among the variables.

**Table 2:** Cox model analysis of factors influencing the risk of impaired ability to perform activities of daily living for five categories of high prevalence chronic diseases (*HR* 95% *CI*).

**Chronic disease**	**45~59 years (*n =* 6,241)**	**60~74 years (*n =* 3,420)**	**75 years and over (*n =* 435)**	**Add up the total (*n =* 10,096)**
**High blood pressure**				
No	1.00	1.00	1.00	1.00
Yes	1.18^*^ (1.01~1.41)	1.27^*^ (1.09~1.48)	1.40^*^ (1.01~1.94)	1.38^*^ (1.24~1.54)
**Dyslipidaemia**				
No	1.00	1.00	1.00	1.00
Yes	1.17 (0.92~1.50)	1.06 (0.84~1.33)	0.58 (0.27~1.22)	1.07 (0.91~1.25)
**Heart disease**				
No	1.00	1.00	1.00	1.00
Yes	1.35^*^ (1.08~1.68)	1.11 (0.92~1.35)	1.08 (0.68~1.74)	1.27^*^ (1.10~1.46)
**Stomach disease**				
No	1.00	1.00	1.00	1.00
Yes	0.99 (0.83~1.17)	1.12 (0.96~1.32)	0.97 (0.63~1.49)	1.02 (0.91~1.41)
**Arthritis**				
No	1.00	1.00	1.00	1.00
Yes	1.45^*^ (1.24~1.6767)	1.34^*^ (1.16~1.56)	0.96 (0.67~1.38)	1.38^*^ (1.25~1.53)

Cox model analyses show results adjusted for age at the follow-up in 2018, region, gender, place of residence, marriage, education, smoking, alcohol consumption, socialization, pain, falls, etc.

^*^P < 0.05.

**Table 3:** Cox model analysis of the factors influencing the number of chronic diseases on the risk of impaired ability to perform daily activities (*HR* 95% *CI*).

**Number of chronic diseases**	**45~59 years (*n =* 6,241)**	**60~74 years (*n =* 3,420)**	**75 years and over (*n =* 435)**	**Add up the total (*n =* 10,096)**
0	1.00	1.00	1.00	1.00
1	1.37^*^ (1.14~1.65)	1.26^*^ (1.03~1.55)	1.02 (0.68~1.52)	1.34^*^ (1.18~1.52)
≥2	1.73^*^ (1.44~2.08)	1.76^*^ (1.45~2.12)	1.07 (0.73~1.56)	1.84^*^ (1.63~2.08)

Cox model analyses show results adjusted for age at the follow-up in 2018, region, gender, place of residence, marriage, education, smoking, alcohol consumption, socialization, pain, falls, etc.

^*^P < 0.05.

In the overall sample, subjects with heart disease had a 27% higher risk of impaired ADLs than people without heart disease (*HR* = 1.27, 95% *CI*: 1.20–1.46), individuals with arthritis had a 38% higher risk of ADL impairment than persons without arthritis (*HR* = 1.38, 95% *CI*: 1.25–1.53), and subjects with hypertension had a 38% higher risk of impaired ADLs compared to subjects without hypertension. Age subgroup analyses of the entire sample revealed that the 45–59-year-old subgroup had a 21% (*HR* = 1.21, 95% *CI*: 1.01–1.45), 37% (*HR* = 1.37, 95% *CI*: 1.09–1.71), and 47% (*HR* = 1.47, 95% *CI*: 1.26–1.71) higher risk of impaired ADL, respectively, due to hypertension, heart disease, and arthritis. In the 60–74-year-old subgroup, hypertension and arthritis increased the risk of impaired ADLs by 28% (*HR* = 1.28, 95% *CI*: 1.10–1.49) and 34% (*HR* = 1.34, 95% *CI*: 1.16–1.55), respectively; in the 75–year-old and older subgroups, suffering from hypertension increased the risk of impaired ADLs by 40% (*HR* = 1.40, 95% *CI*: 1.01–1.94). Moreover, the several cox regression analyses expressed, neither dyslipidaemia nor stomach disease was found to be statistically significantly associated with impaired ADL in the entire sample (Table 2). Compared to those without chronic diseases, the risk of impaired ADL was higher in the overall sample for those with one or more chronic conditions by 34% (*HR* = 1.34, 95% *CI*: 1.18–1.52) and 84% (*HR* = 1.84, 95% *CI*: 1.63–2.08), respectively. The results of the age subgroup analyses indicated that, in the 45–59-year-old subgroup, the probability of impaired ADL was raised by 40% (*HR* = 1.40, 95% *CI*: 1.16–2.59) and 79% (*HR* = 1.79, 95% *CI*: 1.48–2.15), respectively, if one or more chronic diseases were present. Having one or more chronic illnesses increased the likelihood of impaired ADL by 26% (*HR* = 1.26, 95% *CI*: 1.03–1.55) and 77% (*HR* = 1.77, 95% *CI*: 1.47–24.14) in the subgroup of people aged 60–74 years, respectively (Table 3).

## Discussion

This study investigated the relationship between demographic characteristics, health status, types of chronic diseases, and impaired ADL among middle-aged and older adults in China who were 45 years of age and older. It was based on a large-scale population-based survey that used a nationally representative cohort. The results of this research revealed that among China's middle-aged and older population, having multiple chronic diseases, heart disease, arthritis, and hypertension are risk factors for impaired ADL. The likelihood of having impaired ADL also increases with the number of chronic diseases a person possesses.

The results of this present study found that hypertension increases the risk of impaired ADL in middle-aged and older adults, which is consistent with the results of previous studies (18, 19). Moreover, data from the 2011–2015 China Health and Retirement Longitudinal Study (CHARLS) of middle-aged and older adults aged 45 years and older in China found that hypertension was a risk factor for impaired ADL in middle-aged and older adults (18). Further 7-year cohort research carried out in the United States similarly discovered that older persons with hypertension had an increased probability of having impaired ADL (19). About half of China's yearly fatalities from cardiovascular and cerebrovascular disorders are attributable to hypertension, which is well recognized to be extremely hazardous and a significant risk factor for many of these conditions (20). Moreover, studies indicate that the number of fatalities from hypertension and disability-adjusted life years (DALYs) is rising annually (21). The Report on Nutrition and Chronic Disease Status of Chinese Residents (22), states that among Chinese residents who are 18 years of age and older, the prevalence of hypertension is 27.5%; however, the control rate is only around 16.8%. The study's findings indicated that middle-aged and older adults in three distinct age groups: 45–59, 60–74, and 75 years and older, were prone to having impaired ADL due to hypertension. Considering the serious harm and high prevalence of hypertension, it is recommended that middle-aged and older adults with hypertension receive scientifically grounded, comprehensive management that includes early detection, early diagnosis, and early control in order to minimize its effects on ADL impairment.

The results of the study also showed a significant association between the presence of heart disease and impaired ADL in Chinese middle-aged and older adults. Heart disease may have a serious impact on heart function, including ischemia and hypoxia in the myocardial tissues, which in turn leads to a decline in heart function and affects the patients' ADL. Secondly, heart disease may trigger a range of psychological and emotional problems, such as anxiety, depression, and fear, which in turn affect their motivation, willingness, and confidence in performing daily tasks, leading to impaired ADL. This is consistent with the findings of Akasaki et al., who examined older people with and without heart disease using data from community health surveys and discovered a substantial correlation between social vulnerability and heart disease (23). Furthermore, Liperoti et al. conducted a systematic review that yielded similar findings. They searched for relevant papers in PubMed, Web of Science, and Embase. The eligible papers were then subjected to a meta-analysis. The study found that older patients with ischemic heart disease frequently had frailty. Thus, it is critical to take into account the frailty state of older people with ischemic heart disease in order to establish individualized strategies for cardiovascular prevention and treatment (24). Heart diseases, which include congenital heart disease, coronary heart disease, and various forms of heart disease, are collectively referred to as heart disease (25). Ischemia and hypoxia of the myocardial tissues can result from cardiac disease, which can have a major impact on heart function, perhaps even on a normal life. China's aging population continues to grow rapidly, and the burden of heart disease is becoming more and more serious, posing a great challenge to prevention and control (26). Therefore, prevention, diagnosis, treatment, and control of heart disease in middle-aged and older adults are crucial in delaying ADL damage.

Moreover, the investigation's findings indicate that arthritis increases middle-aged and older people's risk of ADL impairment and this is similar to previous findings (27, 28). Arthritis is currently the most disabling disease worldwide (29). Arthritis is an inflammatory disease that occurs in the joints and surrounding tissues of the body and is characterized by redness, swelling, heat, pain, dysfunction, and joint deformity. Common types of arthritis include rheumatoid arthritis, osteoarthritis, reactive arthritis, infectious arthritis, and traumatic arthritis (30, 31). Studies have found that the prevalence of arthritis in China is as high as 50% in people over 60 years of age. The disease is associated with an inability to exert force during movement, and the associated muscles become increasingly atrophied, ultimately leading to impaired daily activities (29). Based on the severity of arthritis, middle-aged and older adults should have early prevention, diagnosis, control, and scientific comprehensive management of arthritis in order to alleviate its impact on ADL impairment.

By examining subgroups of middle-aged and older persons, the research revealed that only the middle-aged group suffered from heart disease and that only the middle-aged and young older adult groups were at higher risk of developing impaired ADL. This clue indicates that younger people with heart disease and arthritis are more likely to have impaired ADL, and previous studies have also reached similar conclusions (32, 33). In the future, the preventive intervention of heart disease and arthritis among the younger population should be further strengthened to reduce the incidence of the diseases in order to delay and control ADL impairment. Heart disease and arthritis were not significantly associated with impaired ADL in the older age group, this may be due to the health-selective effect of death on older age group, and those who manage to live to an advanced age are usually the healthier older people in their cohort. In addition, the study found that middle-aged and older adults with chronic diseases were at increased risk of impaired ADL, and that the risk increased with the number of diseases, which is consistent with previous findings (8). Middle-aged and older adults with impaired functioning and multiple chronic diseases require more health care services and financial support (34), which imposes a significant financial, illness, and emotional burden on individuals, families, and society. Therefore, prevention, diagnosis, treatment, and management of chronic diseases in middle-aged and older adults are necessary to delay ADL impairment.

Unlike previous studies that used a cross-sectional study design (16), the present study used a large-scale population as the study population and adopted a prospective cohort study method to dynamically collect ADL impairment in middle-aged and older adults, to more reasonably evaluate the relationship between suffering from chronic diseases and ADL impairment from a temporal perspective, and to provide reliable evidence for slowing down and controlling ADL impairment in middle-aged and older adults in China. This study also has some limitations; information on the prevalence of chronic diseases and ADL impairment was obtained by self-report, which may be subject to recall bias. However, the present study was able to reduce recall bias to a greater extent by using a scientific and well-recognized scale to measure ADL impairment in middle-aged and older adults and by using self-reported illnesses diagnosed by healthcare institutions as the study factor (14). Therefore, the likelihood of recall bias is small, and the results have a certain degree of reliability.

## Conclusion

In summary, this study found that hypertension is a risk factor for impaired ADL in all age groups, so increasing the rate of awareness, treatment, and control of hypertension is very important for delaying impaired ADL and improving the quality of life in middle-aged and older adults. Additionally, it was discovered in the investigation of the association between the number of chronic diseases and ADL impairment that the presence of chronic diseases increased the risk of ADL impairment in both the middle-aged group (45–59 years old) and the older adult group (60–74 years old), and that the risk increased as the number of diseases increased. Therefore, the prevention and management of chronic diseases in middle-aged and older adults should be strengthened, which is of great public health significance in reducing the risk of ADL impairment.

## Data availability statement

The original contributions presented in the study are included in the article/supplementary material, further inquiries can be directed to the corresponding author.

## Ethics statement

The studies involving human participants were reviewed and approved by Ethical Review Committee of Peking University. The patients/participants provided their written informed consent to participate in this study. The studies were conducted in accordance with the local legislation and institutional requirements. Written informed consent for participation was not required from the participants or the participants' legal guardians/next of kin because the large-scale study organized by the government obtained informed consent from the participants. Written informed consent was not obtained from the individual(s) for the publication of any potentially identifiable images or data included in this article because the large-scale study organized by the government obtained informed consent from the participants.

## Author contributions

ZA: Conceptualization, Data curation, Formal analysis, Methodology, Software, Writing—original draft. CT: Formal analysis, Methodology, Writing—review & editing. XW: Data curation, Writing—review & editing. ST: Funding acquisition, Resources, Supervision, Validation, Visualization, Writing—review & editing. KK: Formal analysis, Methodology, Writing—review & editing.
